# 2-(5,6-Diphenyl-1,2,4-triazin-3-yl)aniline

**DOI:** 10.1107/S1600536812044194

**Published:** 2012-11-03

**Authors:** Mariusz Mojzych, Zbigniew Karczmarzyk, Andrzej Fruziński

**Affiliations:** a Department of Chemistry, Siedlce University, ul. 3 Maja 54, 08-110 Siedlce, Poland; b Department of General and Ecological Chemistry, Technical University, ul. Żeromskiego 115, 90-924 Łódź, Poland

## Abstract

The title compound, C_21_H_16_N_4_, obtained under standard Suzuki cross-coupling conditions, is a model compound in the synthesis and biological activity evaluation of new aza-analogues of sildenafil containing a pyrazolo­[4,3-*e*][1,2,4]triazine system. An N—H⋯N intra­molecular hydrogen bond involving the amino­benzene system and the 1,2,4-triazine moiety helps to establish a near coplanar orientation of the rings with a dihedral angle of 12.04 (4)°, which is believed to be necessary for the biological activity of sildenafil analogues. The 1,2,4-triazine ring is slightly distorted from planarity [r.m.s deviation = 0.0299 (11) Å] and forms dihedral angles of 58.60 (4) and 36.35 (3)° with the pendant phenyl rings. The crystal packing features bifurcated N—H⋯(N,N) hydrogen bonds linking screw-axis-related mol­ecules into chains parallel to the [010] direction and π–π inter­actions, with a centroid–centroid separation of 3.8722 (7) Å and a slippage of 1.412 (3) Å. The crystal studied was a nonmerohedral twin with a ratio of 0.707 (2):0293 (2).

## Related literature

For background information on the activity of sildenafil citrate, see: Terrett *et al.* (1996 ▶); Card *et al.* (2004 ▶). For the synthesis of the title compound, see: Agarwal *et al.* (2010 ▶). For a description of the Cambridge Structural Database, see: Allen (2002 ▶); Bruno *et al.* (2002 ▶).
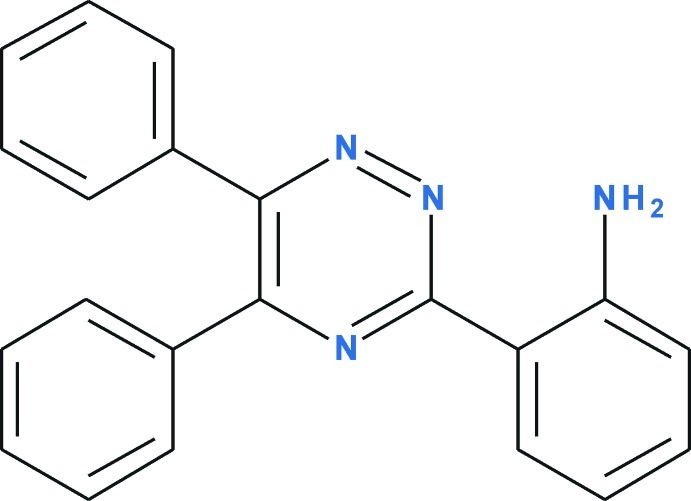


## Experimental

### 

#### Crystal data

C_21_H_16_N_4_
*M* *_r_* = 324.38
Monoclinic, 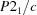

*a* = 11.8797 (3) Å
*b* = 6.0788 (1) Å
*c* = 23.8710 (5) Å
β = 101.489 (1)°
*V* = 1689.29 (6) Å^3^
*Z* = 4
Cu *K*α radiation
μ = 0.61 mm^−1^
*T* = 293 K
0.21 × 0.14 × 0.01 mm

#### Data collection

Bruker APEXII CCD diffractometer
Absorption correction: multi-scan (*SADABS*; Bruker, 2005 ▶) *T*_min_ = 0.906, *T*_max_ = 1.000
4591 measured reflections
3173 independent reflections
2837 reflections with *I* > 2σ(*I*)

#### Refinement

*R*[*F* ^2^ > 2σ(*F* ^2^)] = 0.035
*wR*(*F* ^2^) = 0.103
*S* = 1.05
4591 reflections
275 parameters
All H-atom parameters refined
Δρ_max_ = 0.10 e Å^−3^
Δρ_min_ = −0.13 e Å^−3^

### 

Data collection: *APEX2* (Bruker, 2005 ▶); cell refinement: *SAINT* (Bruker, 2005 ▶); data reduction: *SAINT*; program(s) used to solve structure: *SHELXS97* (Sheldrick, 2008 ▶); program(s) used to refine structure: *SHELXL97* (Sheldrick, 2008 ▶); molecular graphics: *ORTEP-3 for Windows* (Farrugia, 2012 ▶); software used to prepare material for publication: *SHELXL97* and *WinGX* (Farrugia, 2012 ▶).

## Supplementary Material

Crystal structure: contains datablock(s) global, I. DOI: 10.1107/S1600536812044194/lr2082sup1.cif

Structure factors: contains datablock(s) I. DOI: 10.1107/S1600536812044194/lr2082Isup2.hkl

Supplementary material file. DOI: 10.1107/S1600536812044194/lr2082Isup3.cml

Additional supplementary materials:  crystallographic information; 3D view; checkCIF report

## Figures and Tables

**Table 1:** Hydrogen-bond geometry (Å, °)

*D*—H⋯*A*	*D*—H	H⋯*A*	*D*⋯*A*	*D*—H⋯*A*
N7—H72⋯N2	0.94 (2)	2.00 (2)	2.7037 (19)	130 (2)
N7—H71⋯N1^i^	0.93 (3)	2.26 (3)	3.1779 (19)	169 (2)
N7—H71⋯N2^i^	0.93 (3)	2.49 (2)	3.2677 (18)	141.3 (19)

Symmetry codes: (i) 
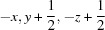
; (ii) 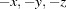.

**Fig. 1.**
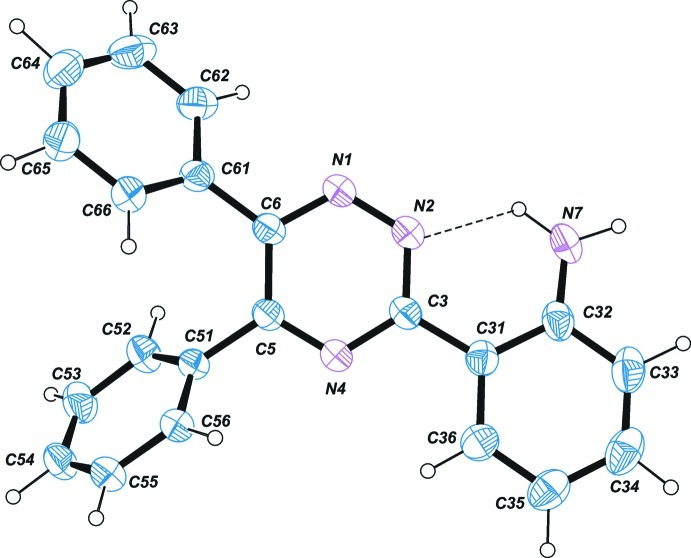
A view of (I) with displacement ellipsoids drawn at the 50% probability level. H atoms are represented as small spheres of arbitrary radii.

**Fig. 2.**
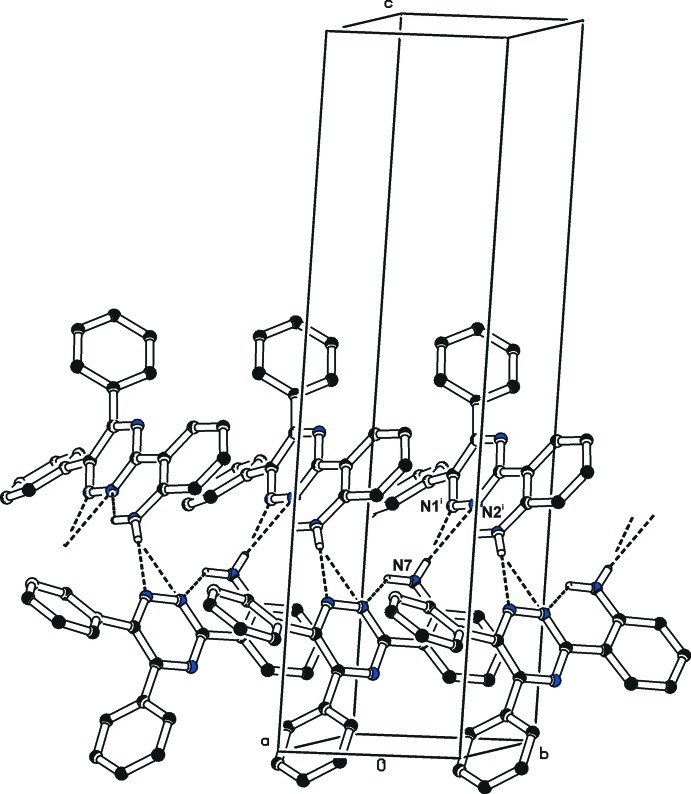
A view of the molecular packing in (I). Dashed lines indicate N—H···N intermolecular hydrogen bond [symmetry code: (i) –x, y + 1/2, –z+1/2].

## supplementary crystallographic information

### Comment

Nowadays, sildenafil citrate (Viagra) is the first orally effective phosphodiesterase type 5 (PDE5) inhibitor available for the treatment of common and important medical problem *e.g.* male erectile dysfunction (MED) (Terrett *et al.*, 1996). The earlier work on crystal structures of the catalytic domains of PDEs with different inhibitors have revealed two common features of inhibitor binding to PDEs: a planar ring structure of the inhibitor and hydrogen bond iteractions with an invariant glutamine residue (Card *et al.*, 2004). With this in mind we have planned a new series of sildenafil analogues with pyrazolo[4,3-*e*][1,2,4]triazine system in which triazine ring nitrogen N1 plays a role of C=O group present in pyrimidinone moiety of sildenafil and ethylamino group in the position 2' of phenyl ring allowed to form intramolecular hydrogen bond between aminophenyl ring and pyrazolotriazine ring system. To clearly define the possibility and the place of intramolecular hydrogen bond formation in the new sildenafil analogues the synthesis and the crystal structure determination of an appropriate model 2-(5,6-diphenyl-1,2,4-triazin-3-yl)aniline, (I), were undertaken.

A search of the Cambridge Structural Database (CSD version 5.33, November 2011; Allen, 2002; Bruno *et al.*, 2002) did not reveal any crystal structures containing the 3-(2-aminophenyl)-1,2,4-triazine structural unit. The structure of the molecule (I) is shown in Fig. 1. One can see that the 3-aminophenyl-1,2,4-triazine system exists in the crystal in the conformation with the torsion angle N2–C3–C31–C32 of -6.86 (17)°. This conformation is forced by the strong N7–H72···N2 intramolecular hydrogen bond (Table 1). The conformation of the 5- and 6-phenyl substituents of the 1,2,4-triazine system in relation to the triazine ring described by the torsion angles N4–C5–C51–C52 of -120.48 (13)° and N1–C6–C61–C62 of 35.44 (15)°, respectively, is forced by the steric effect of these bulky groups in adjacent positions of the heterocyclic system. This strong steric interaction causing the appearance of the strains in the triazine ring results in the distortion of its planarity with the displacements of the triazine atoms from the best plane within 0.0299 (11) Å.

In the crystal structure, the screw-related molecules are linked into chains along the [010] direction by bifurcated N7–H71···N1 and N7–H71···N2 intermolecular hydrogen bonds (Fig. 2) and the methine groups C53—H53 of the inversion-related molecules interact with π-electron system of the aminophenyl ring *via* C—H···π interaction (Table 1). Moreover, nearly coplanar mutual position of the triazine and aminophenyl rings is stabilized by the π–π interaction of these rings in the crystal structure. The π-electron systems of the pairs of triazine and aminophenyl rings belonging to the translation-related molecules overlap each other, with centroid-to-centroid separation of 3.8722 (7) Å between the triazine ring at (*x*, *y*, *z*) and aminophenyl ring at (*x*, -1 + *y*, *z*) and aminophenyl ring at (*x*, *y*, *z*) and triazine ring at (*x*, 1 + *y*, *z*). The π–π distances are 3.2886 (4) and 3.6055 (6) Å, respectively, the angle between overlapping planes is 12.13 (6)° and the slippage is 1.412 (3) Å.

In conclusion, the X-ray investigations of molecule (I) confirmed the assumed possibility of forming the N7–H···N2 intramolecular hydrogen bond stabilizing its *cis* conformation in the crystalline state, analogous to active conformation of sildenafil molecule.

### Experimental

The title compound, (I), was obtained using standard Suzuki cross-coupling conditions (Agarwal *et al.*, 2010), in the reaction of 3-bromo- and 3-chloro-5,6-diphenyl-1,2,4-triazine with 2-aminophenylboronic acid. To a solution of 3-halogeno-5,6-diphenyl-1,2,4-triazine (0.2 mmol) and 2-aminophenylboronic acid (0.22 mmol) in dioxane/water mixture (4:1) (2.5 ml) solution of K_2_CO_3_ (0.6 mmol) in 1 ml of water and Pd(PPh_3_)_4_ were added. The reaction mixture was stirred at 70°C for 12 h. After that time the solution was diluted with H_2_O (1.5 ml), and then the product was extracted three times with CH_2_Cl_2_ (3x2mL). The combined organic layer was dried over Na_2_SO_4_ and the solvent was removed *in vacuo*. The crude product was then subjected to column chromatography using CH_2_Cl_2_:hexane (4:1) as eluent. mp 161°C. Crystals suitable for X-ray diffraction analysis were grown by slow evaporation of an ethanol solution.

### Refinement

The structure of (I) was refined as a nonmerohedral twin using 4591 reflections in the HKLF 5 file format and a BASF parameter of 0.70752 in *SHELXL97* (Sheldrick, 2008). All H atoms were located from difference electron-density maps and their coordinates were refined with isotropic displacement parameters taken as 1.5 times those of the respective parent atoms.

### Crystal data

**Table:**

C_21_H_16_N_4_	*F*(000) = 680
*M**_r_* = 324.38	*D*_x_ = 1.275 Mg m^−^^3^
Monoclinic, *P*2_1_/*c*	Melting point: 434 K
Hall symbol: -P 2ybc	Cu *K*α radiation, λ = 1.54178 Å
*a* = 11.8797 (3) Å	Cell parameters from 241 reflections
*b* = 6.0788 (1) Å	θ = 8.2–35.4°
*c* = 23.8710 (5) Å	µ = 0.61 mm^−^^1^
β = 101.489 (1)°	*T* = 293 K
*V* = 1689.29 (6) Å^3^	Prism, yellow
*Z* = 4	0.21 × 0.14 × 0.01 mm

### Data collection

**Table:**

Bruker APEXII CCD diffractometer	3173 independent reflections
Radiation source: fine-focus sealed tube	2837 reflections with *I* > 2σ(*I*)
Graphite monochromator	*R*_int_ = 0.000
ω scans	θ_max_ = 69.9°, θ_min_ = 3.8°
Absorption correction: multi-scan (*SADABS*; Bruker, 2005)	*h* = −14→14
*T*_min_ = 0.906, *T*_max_ = 1.000	*k* = 0→7
4591 measured reflections	*l* = 0→29

### Refinement

**Table:**

Refinement on *F*^2^	Primary atom site location: structure-invariant direct methods
Least-squares matrix: full	Secondary atom site location: difference Fourier map
*R*[*F*^2^ > 2σ(*F*^2^)] = 0.035	Hydrogen site location: difference Fourier map
*wR*(*F*^2^) = 0.103	All H-atom parameters refined
*S* = 1.05	*w* = 1/[σ^2^(*F*_o_^2^) + (0.0591*P*)^2^ + 0.0858*P*] where *P* = (*F*_o_^2^ + 2*F*_c_^2^)/3
4591 reflections	(Δ/σ)_max_ < 0.001
275 parameters	Δρ_max_ = 0.10 e Å^−^^3^
0 restraints	Δρ_min_ = −0.13 e Å^−^^3^

### Special details

**Table:**

Experimental. ^1^H-NMR (400 mHz, DMSO) *δ*: 6.85 (*d*, *J* = 7.2 Hz, 1H), 7.31 (*t*, *J* = 8.0 Hz, 1H), 7.35–7.45 (*m*, 6H), 7.60 (*d*, *J* = 8.0 Hz, 2H), 7.65 (*d*, *J* = 8.0 Hz, 2H), 8.61 (*d*, *J* = 8.0 Hz, 1H). ^13^C-NMR (100 MHz, DMSO) *δ*:167.68, 164.37, 162.95, 155.22, 153.98, 147.70, 135.91, 135.47, 132.71, 130.89, 130.77, 130.71, 129.82, 129.51, 129.36, 128.81, 128.61, 128.55, 117.98.
Geometry. All e.s.d.'s (except the e.s.d. in the dihedral angle between two l.s. planes) are estimated using the full covariance matrix. The cell e.s.d.'s are taken into account individually in the estimation of e.s.d.'s in distances, angles and torsion angles; correlations between e.s.d.'s in cell parameters are only used when they are defined by crystal symmetry. An approximate (isotropic) treatment of cell e.s.d.'s is used for estimating e.s.d.'s involving l.s. planes.
Refinement. Refinement of *F*^2^ against ALL reflections. The weighted *R*-factor *wR* and goodness of fit *S* are based on *F*^2^, conventional *R*-factors *R* are based on *F*, with *F* set to zero for negative *F*^2^. The threshold expression of *F*^2^ > σ(*F*^2^) is used only for calculating *R*-factors(gt) *etc*. and is not relevant to the choice of reflections for refinement. *R*-factors based on *F*^2^ are statistically about twice as large as those based on *F*, and *R*- factors based on ALL data will be even larger.

### Fractional atomic coordinates and isotropic or equivalent isotropic displacement parameters (Å^2^)

**Table:**

	*x*	*y*	*z*	*U*_iso_*/*U*_eq_	
N1	0.18725 (9)	−0.11023 (15)	0.18454 (4)	0.0576 (2)	
N2	0.10666 (8)	0.04609 (16)	0.17863 (4)	0.0597 (2)	
N4	0.14421 (8)	0.14537 (15)	0.08844 (4)	0.0526 (2)	
N7	−0.07122 (15)	0.2512 (2)	0.21174 (7)	0.0864 (5)	
H71	−0.114 (2)	0.294 (3)	0.2383 (11)	0.130*	
H72	−0.025 (2)	0.125 (4)	0.2158 (10)	0.130*	
C3	0.08941 (9)	0.1743 (2)	0.13234 (4)	0.0514 (2)	
C5	0.21940 (9)	−0.01610 (17)	0.09270 (4)	0.0492 (2)	
C6	0.24565 (9)	−0.14379 (17)	0.14325 (4)	0.0500 (2)	
C31	0.00852 (9)	0.35952 (19)	0.12884 (5)	0.0547 (3)	
C32	−0.06168 (10)	0.3956 (2)	0.16965 (5)	0.0637 (3)	
C33	−0.12606 (12)	0.5930 (3)	0.16472 (6)	0.0777 (4)	
H33	−0.1748 (17)	0.619 (3)	0.1937 (8)	0.117*	
C34	−0.12449 (16)	0.7395 (2)	0.12196 (9)	0.0822 (5)	
H34	−0.1621 (18)	0.878 (4)	0.1199 (9)	0.123*	
C35	−0.06099 (14)	0.6990 (3)	0.07991 (8)	0.0766 (4)	
H35	−0.0576 (19)	0.808 (3)	0.0485 (10)	0.115*	
C36	0.00405 (11)	0.5109 (2)	0.08408 (6)	0.0641 (3)	
H36	0.0494 (15)	0.481 (3)	0.0544 (7)	0.096*	
C51	0.27044 (9)	−0.05244 (18)	0.04130 (4)	0.0511 (2)	
C52	0.25810 (15)	−0.25262 (19)	0.01303 (7)	0.0654 (4)	
H52	0.2176 (15)	−0.371 (3)	0.0296 (8)	0.098*	
C53	0.30015 (16)	−0.2788 (3)	−0.03670 (7)	0.0752 (4)	
H53	0.2884 (17)	−0.418 (3)	−0.0560 (8)	0.113*	
C54	0.35701 (13)	−0.1102 (3)	−0.05686 (5)	0.0746 (4)	
H54	0.3858 (16)	−0.127 (3)	−0.0913 (8)	0.112*	
C55	0.37091 (12)	0.0887 (3)	−0.02854 (6)	0.0714 (4)	
H55	0.412 (2)	0.210 (3)	−0.0428 (10)	0.107*	
C56	0.32644 (11)	0.1187 (2)	0.02024 (5)	0.0606 (3)	
H56	0.3355 (18)	0.266 (2)	0.0396 (9)	0.091*	
C61	0.33740 (11)	−0.3132 (2)	0.15615 (5)	0.0551 (3)	
C62	0.32080 (13)	−0.4980 (2)	0.18810 (5)	0.0687 (3)	
H62	0.2476 (16)	−0.510 (3)	0.2016 (8)	0.103*	
C63	0.40801 (19)	−0.6518 (2)	0.20237 (6)	0.0890 (5)	
H63	0.393 (2)	−0.782 (3)	0.2224 (12)	0.133*	
C64	0.51152 (18)	−0.6245 (3)	0.18542 (7)	0.0932 (5)	
H64	0.575 (3)	−0.733 (3)	0.1959 (13)	0.140*	
C65	0.52850 (15)	−0.4423 (3)	0.15449 (6)	0.0865 (5)	
H65	0.6019 (19)	−0.420 (3)	0.1395 (9)	0.130*	
C66	0.44224 (14)	−0.2872 (3)	0.13971 (6)	0.0682 (3)	
H66	0.4576 (16)	−0.156 (3)	0.1189 (8)	0.102*	

### Atomic displacement parameters (Å^2^)

**Table:**

	*U*^11^	*U*^22^	*U*^33^	*U*^12^	*U*^13^	*U*^23^
N1	0.0658 (6)	0.0613 (5)	0.0496 (5)	0.0045 (4)	0.0205 (4)	0.0065 (4)
N2	0.0642 (6)	0.0681 (6)	0.0514 (5)	0.0073 (5)	0.0225 (4)	0.0050 (4)
N4	0.0578 (5)	0.0561 (5)	0.0460 (5)	0.0022 (4)	0.0153 (4)	0.0013 (4)
N7	0.0968 (11)	0.1108 (12)	0.0611 (8)	0.0297 (7)	0.0386 (7)	0.0003 (6)
C3	0.0519 (6)	0.0588 (6)	0.0452 (5)	−0.0024 (5)	0.0135 (4)	−0.0032 (5)
C5	0.0530 (5)	0.0511 (5)	0.0451 (5)	−0.0014 (4)	0.0136 (4)	−0.0003 (4)
C6	0.0563 (6)	0.0514 (6)	0.0440 (5)	−0.0031 (4)	0.0144 (4)	0.0006 (4)
C31	0.0508 (6)	0.0607 (6)	0.0507 (6)	0.0018 (5)	0.0058 (4)	−0.0076 (5)
C32	0.0571 (6)	0.0798 (8)	0.0524 (6)	0.0077 (6)	0.0062 (5)	−0.0177 (5)
C33	0.0649 (8)	0.0954 (10)	0.0671 (8)	0.0208 (7)	−0.0006 (6)	−0.0267 (7)
C34	0.0694 (10)	0.0752 (10)	0.0900 (13)	0.0193 (6)	−0.0132 (9)	−0.0201 (7)
C35	0.0697 (9)	0.0695 (7)	0.0828 (10)	0.0051 (7)	−0.0039 (7)	0.0023 (8)
C36	0.0578 (7)	0.0660 (7)	0.0659 (7)	0.0021 (6)	0.0057 (5)	0.0011 (6)
C51	0.0525 (6)	0.0593 (6)	0.0426 (5)	0.0065 (5)	0.0121 (4)	0.0044 (4)
C52	0.0730 (10)	0.0680 (8)	0.0584 (8)	−0.0055 (5)	0.0212 (7)	−0.0080 (5)
C53	0.0830 (10)	0.0850 (9)	0.0607 (9)	0.0037 (7)	0.0216 (7)	−0.0181 (6)
C54	0.0779 (9)	0.1029 (11)	0.0487 (6)	0.0177 (8)	0.0259 (6)	0.0037 (6)
C55	0.0747 (8)	0.0833 (8)	0.0635 (7)	0.0113 (7)	0.0315 (6)	0.0178 (6)
C56	0.0680 (7)	0.0609 (6)	0.0571 (6)	0.0083 (5)	0.0225 (5)	0.0090 (5)
C61	0.0674 (7)	0.0539 (5)	0.0433 (5)	0.0046 (6)	0.0093 (5)	−0.0016 (5)
C62	0.0915 (9)	0.0567 (7)	0.0547 (6)	−0.0009 (6)	0.0064 (6)	0.0018 (5)
C63	0.1393 (15)	0.0572 (8)	0.0612 (8)	0.0166 (8)	−0.0020 (8)	0.0044 (6)
C64	0.1146 (13)	0.0931 (11)	0.0642 (8)	0.0476 (10)	−0.0003 (8)	−0.0045 (8)
C65	0.0844 (10)	0.1098 (11)	0.0645 (8)	0.0360 (9)	0.0129 (7)	0.0006 (8)
C66	0.0714 (9)	0.0806 (8)	0.0543 (7)	0.0163 (7)	0.0165 (6)	0.0052 (6)

### Geometric parameters (Å, º)

**Table:**

N1—C6	1.3293 (13)	C51—C56	1.3824 (16)
N1—N2	1.3363 (13)	C51—C52	1.3850 (16)
N2—C3	1.3344 (14)	C52—C53	1.386 (2)
N4—C5	1.3174 (13)	C52—H52	0.990 (17)
N4—C3	1.3509 (13)	C53—C54	1.367 (2)
N7—C32	1.3559 (19)	C53—H53	0.96 (2)
N7—H71	0.92 (3)	C54—C55	1.379 (2)
N7—H72	0.94 (2)	C54—H54	0.957 (19)
C3—C31	1.4717 (16)	C55—C56	1.3831 (17)
C5—C6	1.4164 (14)	C55—H55	0.98 (2)
C5—C51	1.4893 (14)	C56—H56	1.003 (15)
C6—C61	1.4865 (16)	C61—C66	1.3874 (19)
C31—C36	1.4029 (17)	C61—C62	1.3936 (17)
C31—C32	1.4200 (16)	C62—C63	1.387 (2)
C32—C33	1.4152 (19)	C62—H62	0.989 (18)
C33—C34	1.358 (2)	C63—C64	1.379 (3)
C33—H33	1.000 (19)	C63—H63	0.96 (2)
C34—C35	1.393 (3)	C64—C65	1.368 (3)
C34—H34	0.95 (2)	C64—H64	0.99 (3)
C35—C36	1.372 (2)	C65—C66	1.385 (2)
C35—H35	1.01 (2)	C65—H65	1.02 (2)
C36—H36	0.988 (17)	C66—H66	0.973 (19)
			
C6—N1—N2	119.97 (9)	C52—C51—C5	120.71 (10)
C3—N2—N1	119.44 (9)	C51—C52—C53	119.81 (12)
C5—N4—C3	117.47 (9)	C51—C52—H52	117.1 (10)
C32—N7—H71	117.6 (14)	C53—C52—H52	123.1 (10)
C32—N7—H72	118.4 (13)	C54—C53—C52	120.20 (14)
H71—N7—H72	123 (2)	C54—C53—H53	121.8 (11)
N2—C3—N4	123.24 (10)	C52—C53—H53	118.0 (11)
N2—C3—C31	118.99 (9)	C53—C54—C55	120.29 (12)
N4—C3—C31	117.75 (10)	C53—C54—H54	120.6 (12)
N4—C5—C6	120.33 (9)	C55—C54—H54	119.1 (12)
N4—C5—C51	115.34 (9)	C54—C55—C56	119.98 (13)
C6—C5—C51	124.33 (9)	C54—C55—H55	120.5 (12)
N1—C6—C5	119.27 (10)	C56—C55—H55	119.6 (12)
N1—C6—C61	114.97 (9)	C55—C56—C51	119.95 (12)
C5—C6—C61	125.74 (9)	C55—C56—H56	118.8 (11)
C36—C31—C32	118.88 (11)	C51—C56—H56	121.2 (11)
C36—C31—C3	118.05 (10)	C66—C61—C62	118.64 (13)
C32—C31—C3	123.04 (10)	C66—C61—C6	121.68 (11)
N7—C32—C33	119.15 (12)	C62—C61—C6	119.60 (11)
N7—C32—C31	123.76 (11)	C63—C62—C61	119.86 (15)
C33—C32—C31	117.09 (13)	C63—C62—H62	122.1 (10)
C34—C33—C32	122.06 (14)	C61—C62—H62	118.0 (10)
C34—C33—H33	120.7 (12)	C64—C63—C62	120.86 (15)
C32—C33—H33	117.2 (12)	C64—C63—H63	120.9 (15)
C33—C34—C35	121.08 (13)	C62—C63—H63	118.1 (15)
C33—C34—H34	122.9 (12)	C65—C64—C63	119.45 (15)
C35—C34—H34	116.0 (12)	C65—C64—H64	119.1 (15)
C36—C35—C34	118.24 (15)	C63—C64—H64	121.5 (15)
C36—C35—H35	119.8 (12)	C64—C65—C66	120.48 (17)
C34—C35—H35	121.8 (12)	C64—C65—H65	121.9 (12)
C35—C36—C31	122.48 (13)	C66—C65—H65	117.5 (12)
C35—C36—H36	118.7 (10)	C65—C66—C61	120.72 (14)
C31—C36—H36	118.8 (10)	C65—C66—H66	118.8 (11)
C56—C51—C52	119.73 (11)	C61—C66—H66	120.4 (11)
C56—C51—C5	119.50 (10)		
			
C6—N1—N2—C3	−3.18 (16)	C32—C31—C36—C35	3.59 (18)
N1—N2—C3—N4	4.71 (17)	C3—C31—C36—C35	−174.22 (12)
N1—N2—C3—C31	−174.12 (10)	N4—C5—C51—C56	56.74 (14)
C5—N4—C3—N2	−1.09 (16)	C6—C5—C51—C56	−124.24 (12)
C5—N4—C3—C31	177.74 (10)	N4—C5—C51—C52	−120.48 (13)
C3—N4—C5—C6	−3.78 (15)	C6—C5—C51—C52	58.55 (16)
C3—N4—C5—C51	175.29 (10)	C56—C51—C52—C53	−1.1 (2)
N2—N1—C6—C5	−1.55 (16)	C5—C51—C52—C53	176.07 (14)
N2—N1—C6—C61	176.68 (10)	C51—C52—C53—C54	2.2 (3)
N4—C5—C6—N1	5.17 (16)	C52—C53—C54—C55	−1.3 (2)
C51—C5—C6—N1	−173.81 (10)	C53—C54—C55—C56	−0.5 (2)
N4—C5—C6—C61	−172.85 (10)	C54—C55—C56—C51	1.5 (2)
C51—C5—C6—C61	8.17 (17)	C52—C51—C56—C55	−0.68 (19)
N2—C3—C31—C36	170.85 (10)	C5—C51—C56—C55	−177.92 (11)
N4—C3—C31—C36	−8.04 (16)	N1—C6—C61—C66	−141.19 (12)
N2—C3—C31—C32	−6.86 (17)	C5—C6—C61—C66	36.90 (18)
N4—C3—C31—C32	174.25 (10)	N1—C6—C61—C62	35.44 (15)
C36—C31—C32—N7	174.38 (14)	C5—C6—C61—C62	−146.47 (11)
C3—C31—C32—N7	−7.92 (19)	C66—C61—C62—C63	−0.48 (19)
C36—C31—C32—C33	−4.54 (17)	C6—C61—C62—C63	−177.22 (11)
C3—C31—C32—C33	173.16 (11)	C61—C62—C63—C64	0.1 (2)
N7—C32—C33—C34	−176.88 (15)	C62—C63—C64—C65	0.5 (2)
C31—C32—C33—C34	2.1 (2)	C63—C64—C65—C66	−0.7 (2)
C32—C33—C34—C35	1.6 (2)	C64—C65—C66—C61	0.3 (2)
C33—C34—C35—C36	−2.7 (2)	C62—C61—C66—C65	0.3 (2)
C34—C35—C36—C31	0.1 (2)	C6—C61—C66—C65	176.97 (13)

### Hydrogen-bond geometry (Å, º)

Cg - the centroid of the C31-C36 phenyl ring.

**Table:**

*D*—H···*A*	*D*—H	H···*A*	*D*···*A*	*D*—H···*A*
N7—H72···N2	0.94 (2)	2.00 (2)	2.7037 (19)	130 (2)
N7—H71···N1^i^	0.93 (3)	2.26 (3)	3.1779 (19)	169 (2)
N7—H71···N2^i^	0.93 (3)	2.49 (2)	3.2677 (18)	141.3 (19)
C53—H53···*Cg*^ii^	0.96 (2)	2.99 (2)	3.5888 (19)	122.0 (14)

Symmetry codes: (i) −*x*, *y*+1/2, −*z*+1/2; (ii) −*x*, −*y*, −*z*.

## References

[bb1] Agarwal, P. K., Saifuddin, M. & Kundu, B. (2010). *Tetrahedron*, **66**, 862–870.

[bb2] Allen, F. H. (2002). *Acta Cryst.* B**58**, 380–388. 10.1107/s0108768102003890 12037359

[bb3] Bruker (2005). *APEX2*, *SAINT* and *SADABS* Bruker AXS Inc., Madison, Wisconsin, USA.

[bb4] Bruno, I. J., Cole, J. C., Edgington, P. R., Kessler, M., Macrae, C. F., McCabe, P., Pearson, J. & Taylor, R. (2002). *Acta Cryst.* B**58**, 389–397. 10.1107/s0108768102003324 12037360

[bb5] Card, G. L., England, B. P., Suzuki, Y., Fong, D., Powell, B., Lee, B., Luu, C., Tabrizizad, M., Gillette, S., Ibrahim, P. N., Artis, D. R., Bollag, G., Milburn, M. V., Kim, S. H., Schlessinger, J. & Zhang, K. Y. (2004). *Structure*, **12**, 2233–2247. 10.1016/j.str.2004.10.004 15576036

[bb6] Farrugia, L. J. (2012). *J. Appl. Cryst.* **45**, 849–854.

[bb7] Sheldrick, G. M. (2008). *Acta Cryst.* A**64**, 112–122. 10.1107/S0108767307043930 18156677

[bb8] Terrett, N. K., Bell, A. S., Brown, D. & Ellis, P. (1996). *Bioorg. Med. Chem. Lett.* **6**, 1819–1824.

